# Drug Resistance and Novel Therapies in Cancers

**DOI:** 10.3390/cancers12102929

**Published:** 2020-10-12

**Authors:** Zhixiang Wang

**Affiliations:** Signal Transduction Research Group, Department of Medical Genetics, Faculty of Medicine and Dentistry, University of Alberta, Edmonton, AB T6G 2H7, Canada; zwang@ualberta.ca

Cancer is among the leading causes of mortality in the world, accounting for an estimated 9.6 million deaths in 2018. Despite significant advances in cancer therapy, drug resistance remains the principal limiting factor in achieving cures in patients with cancers. Scientific communities have been working intensively to understand the molecular mechanisms underlying drug resistance and to develop novel therapies to overcome drug resistance. Under these circumstances, *Cancers* launched the topic collection “Drug Resistance and Novel Therapies in Cancers” in the second half of 2018.

In the last four months of 2018, eight cutting-edge research articles and comprehensive review papers had been published in this topic collection. One research article explored the possibility of increasing the sensitivity of triple negative breast cancer (TNBC) cells to hormonal therapy [1]. This is a novel approach and yielded interesting data. The research showed that in TNBC cells, histone deacetylases (HDAC) 1 and 2 were less expressed, whereas HDACs 4 and 6 were overexpressed. Interestingly, treatment with epigenetic modifiers including suberoylanilide hydroxamic acid (SAHA) and 5-aza-dc resulted in an increase in the expression of Estrogen Receptor-α (Erα) and HER2/ERBB2 and in the sensitivity of TNBC cells to tamoxifen. This research provided the rationale for the use of epigenetic modifiers to enhance the response of TNBC to hormonal therapy through upregulation of Erα.

Two research articles were related to melanoma [2,3]. One article was focused on targeting MAPK and the p53 pathway simultaneously to treat cutaneous melanoma [2]. This research revealed that ATM-dependent DUSP6 modulation of p53 is involved in synergistic targeting of MAPK and the p53 pathway by MEK inhibitor trametinib and MDM2 inhibitor nutlin-3/RG7388/HDM201 in cutaneous melanoma. The identification of this novel DUSP6 mechanism in BRAFV600E and p53WT melanoma cells provided a novel therapeutic target for treating melanoma. Another article aimed to understand the molecular mechanisms underlying the resistance of melanoma cells to targeted therapy [3]. It was revealed by this research that the resistant melanoma cells acquire transcriptomic similarities with human melanoblasts. This research demonstrated that in vitro models of human melanoblasts could be used to study drug resistance in melanoma. 

Anther research aimed to indirectly target rapamycin complex 1 (mTORC1) hyperactivity in cancers by exploiting homeostatic vulnerabilities within Tuberous Sclerosis Complex 2 (TSC2)-deficient cells [4]. Both chloroquine (an autophagy inhibitor) and nelfinavir (an ER stress inducer) selectively enhance cell death in mTORC1 hyperactive cells. The effect of their combination was examined in both TSC2-deficient cells and cells with wild type TSC2. The data demonstrated a critical vulnerability in cancer cells with aberrant signaling through the TSC2-mTORC1 pathway that lacked flexibility in homeostatic pathways. Thus, combined nelfinavir and mefloquine treatment could be a potential therapy for cancers with mTORC1 hyperactivity.

Ionizing radiation (IR) has been an important treatment for colorectal cancer (CRC). However, IR activates nuclear factor kappa B (NF-κB) that causes radio-resistance and stimulates matrix metalloproteinase (MMP)-2/-9, which promote tumor migration and invasion. The research by Sugano et al. [5] examined the combined effects of nafamostat mesilate (FUT175), a synthetic serine protease inhibitor that inhibits NF-κB activation, and IR on cell proliferation, migration, and invasion of CRC cells. They showed that FUT175 enhances the antitumor effect of radiotherapy through downregulation of NF-κB and reduces IR-induced tumor invasiveness by directly inhibiting MMP-2/-9 in CRC cells. Thus, addition of FUT175 might improve the efficacy of radiotherapy in patients with CRC.

Three published reviews covered three important areas of cancer therapy. One review article covered the latest advances in a hepatocellular carcinoma therapy that is a rapidly evolving [6]. While the development of novel targeted therapy had been fruitless from 2007 to 2016, four new drugs—regorafenib, lenvatinib, cabozantinib, and ramucirumab—emerged successfully from clinical trials and became available clinically in the following two years. The review also summarized and analyzed the recent advances in terms of immune checkpoint inhibitors. Trastuzumab and pertuzumab are two HER2-targeted monoclonal antibodies used as adjuvant therapy in combination with docetaxel to treat metastatic HER2-positive breast cancer. The mechanisms underlying the synergism of the two antibodies are not well-defined. A review article by Nami et al. [7] examined and analyzed findings and hypotheses regarding the action and synergism of trastuzumab and pertuzumab. The authors proposed a model of synergism based on available information. The third review focused on the therapeutic perspectives of NF-κB signaling in targeting tumor cells by oncolytic viruses (OV) [8].
